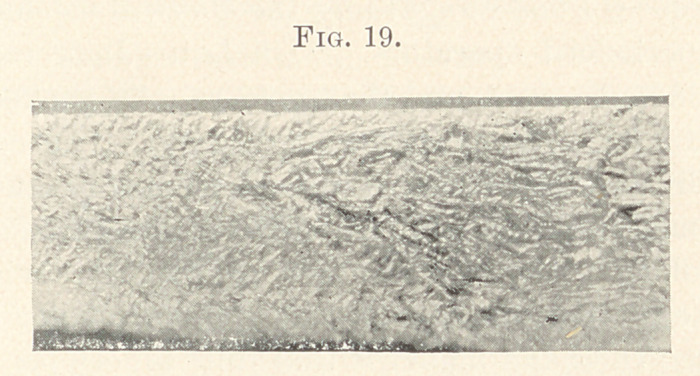# A Method of Inserting Gold Fillings with the Use of Hand-Burnishers, as Practised Exclusively for Seventeen Years

**Published:** 1895-06

**Authors:** Henry F. Libby

**Affiliations:** Boston, Mass.


### International Dental Journal.

Vol. XVI. June, 1895. No. 6.

### Original Communications.[1]

### A METHOD OF INSERTING GOLD FILLINGS WITH THE USE OF HAND-BURNISHERS, AS PRACTISED EXCLUSIVELY FOR SEVENTEEN YEARS.[2]

BY DR. HENRY F. LIBBY, BOSTON, MASS.

In attempting to place before you to-night an experience covering so many years, I must necessarily begin with the earliest incidents, so that you may be familiar with all its motives and results.

Twenty-one years ago, Dr. W. H. Atkinson, of New York City, visited the Harvard Dental Infirmary for the express purpose of instructing the students upon the use of the hand-mallet for filling teeth with gold, and endeavored to prove that this method was the best for preserving them.

A few weeks later, Dr. T. D. Shumway, of Plymouth, Massachusetts, was introduced to give a clinic before the students of the same class, with an original method of using ivory points for "packing gold fillings," and claimed that this would give the best results in saving teeth.

At this early period of my chosen profession I stood face to face with two extreme advocates, involving great principles. Therefore

[1] The editor and publishers are not responsible for the views of authors of papers published in this department, nor for any claim to novelty, or otherwise, that may be made by them. No papers will be received for this department that have appeared in any other journal published in the country.

[2] Read before the American Academy of Dental Science, December 5, 1894.

I concluded to visit Professor Thomas H. Chandler, of Boston, then instructor at the school, to see if he could not throw some light upon this mystery. I found him in a dilemma concerning these theories, but kind and glad to direct me to an apparently safe method to pursue. He advised me to purchase the Varney set of pluggers, and to use hand-pressure with cohesive gold; still, he considered the automatic mallet safe in experienced hands. After this interview I abandoned all thought of adopting Dr. Atkinson's opinions, and experimented upon infirmary patients with ivory points that I made myself, copying as near as possible Dr. Shumway's, but introducing more delicate points to reach small and difficult cavities that we are constantly called upon to treat.

Here was where I lost all hope of Dr. Shumway's method becoming practical. The pressure required to condense the gold properly would break the point, and for contouring, the delicacy of the edge-strength of the instrument could not be relied upon, and I gave up its use altogether.

However, gentlemen, personally I give Dr. Shumway the greatest credit for the suggestion that gold can be manipulated without the use of serrated points, although he asserts that gold cannot be burnished with ivory points. To quote his own words from a paper on ivory points, read before the Massachusetts Dental Society, February, 1872,—

"Ivory points are not pluggers, but are designed for packing gold; they are not burnishers, for gold cannot be burnished with ivory; no instrument can be constructed that will admit of direct application in all sorts of cavities. A smooth-faced plugger, be it ever so carefully formed, if it be made of steel, will slip under the force necessary to consolidate gold-foil. A burnished surface is not favorable to aggregation."

Please pardon me for occupying so much time with these quotations, but I would like to have it understood in what attitude I stand with regard to Dr. Shumway's method.

I am here to give the results of twenty-one years' experimental work, seventeen of which have been devoted to burnishing one piece of gold upon another with steel instruments until a filling is matured.

I was deeply impressed with the opinions of Drs. Shumway and Chandler, notwithstanding all the professors of the school at that time advocated the use of the automatic mallet. The next four years I experimented studiously with hand-pressure, using

Varney points and the automatic mallet. During that period I found a decided preference in hand-pressure.

Now I will show you upon the screen the instruments used during these years, and you will discover in these series of slides that the fundamental principle of success lies with the instruments.

Fig. 1.

In this set of Varney's pluggers (Fig. 1) the two most favorite points were this long and rather delicate obtuse foot-plugger, and its companion, a shorter foot-plugger. These I could manipulate with greater freedom in the incisors and cuspids than other shapes, using the back of the points as well as the face.

It was perfectly surprising how attached I became to this pair of points, and probably should have been operating with them to-day had I not discovered their incapacity to do better work. Possibly this feeling of sentiment arose from the fact that the rapidly-increasing confidence in the burnishing process began with these instruments.

It came about in this way: the slight serrations becoming worn by constant use, I found the gold during the packing process showed a burnished appearance, yet cohered perfectly by passing the instrument across the filling with an upward and downward stroke as the case required. This occurred during the latter part of the first four years of my practice.

The flat burnisher, commonly used after the filling is completed, followed these instruments. Here was the beginning of this method which gave me such anxiety that I shall never forget its burden.

Yet how beautiful was the relief when my patients' annual and semiannual visits rewarded me with few failures. Where were these failures? at the cervical borders, and why? because the instruments were ill-adapted to reach this most vital spot.

The angles of the points were not compatible with the form of the cavity. The handles were too delicate to be held with sufficient strength for condensing the gold properly. Hence, the impact was not perfect. Did the gold cleave or not cohere? No! why, then, these failures?

I was convinced that they were due to the instruments; therefore I purchased the Harvard set, which you now see (Fig. 2). Then I

Fig. 2.

began the use of the corkscrew form of points for burnishing, which has proved the greatest blessing to the patients' comfort and personal gratification.

These are the forms that were chosen to perform the work of the next five years. I immediately began to improve upon the fillings of the incisors and cuspids after these instruments were introduced, which was evidence to my mind that the corkscrew form was an advance in these particular cavities. Now failures appeared at the cervical walls of the bicuspids and molars; the same unfortunate conditions existed here as with the Varney points,—the inadequateness of the instruments to reach the deep distal and mesial cavities of these last-named teeth; also the same difficulties appeared regarding the handles. I felt the necessity of getting more purchase near the point, and therefore the idea came to me of short-shanked instruments. It was then I began to make patterns of instruments and shape of handles as my experience seemed to demand.

The following (Fig. 3) will show how crude and clumsy was my first design.

The handles of steel were needlessly heavy, and tapered at an inconvenient place, yet they were an improvement upon the Harvard set. I operated with these for the next four years, using the obtuse, round points for packing soft foil at the base of the cavities of bicuspids and molars, and completing the fillings by burnishing with a longer pair of corkscrew points, designed by Dr. E. E. Hopkins, of Boston.

Fig. 3.

With these points I was able to reach any desired depth, and contoured with these two flat obtuse burnishers. Now I have had an experience of nine years with this method. My success was established as far as cohesion of gold by burnishing was concerned, even under the difficulties that pursued me with imperfect instruments. However, I could not get close enough to my working point, even with this set, without great fatigue to my hand, and I saw the necessity of larger handles near the points. These were not practical in steel, and the thought of wooden ones came to me. I designed these instruments, with short shanked points, to enter a solid piece of metal that penetrated the handle. (Fig. 4.)

Fig. 4.

The matter of wooden handles and sockets is old to the profession, my modifications simply being shortening the points and shaping the handles to suit my taste.

I will not dwell upon this set, only to say that the hexagonal handles were a mere fancy which I regretted after practical use: the corners cut my hand and hardened the cushions of my finger-tips.

The most important features were the introduction of very long well-curved corkscrew points, varying in sizes. With the largest of these I was able to pack with ease and satisfaction soft foil at the cervical walls of molars and bicuspids. The success of arresting decay at this most vulnerable point with these instruments was assured after a test of four years, which I considered a proper length of time to test any gold work.

I had now arrived at a point where I felt the solid earth beneath my feet, and did not have doubtful thoughts when attempting the most difficult contouring, building out lost and broken corners, showing abraded and worn masticating surfaces, with perfect confidence.

It is interesting to note that during all these years I have used only one manufacturer's gold, both in soft and cohesive foils, and by this method was better able to determine where the failures existed.

I was restless with these handles, and saw that the point could be shortened still more to advantage.

Before entering upon the last set of instruments I want to express my gratitude and thanks to Mr. Frank K. Hesse, manager of the manufacturing department at the Codman and Shurtleff house. He has been untiring in his efforts to follow out my wishes, and it is only with the patient care of such a person that we can accomplish our purpose.

If you can be patient, I would like to go into minute detail in describing the last results of this complete set of thirteen instruments, which I will pass around and also show on the screen.

Fig. 5

First, you will observe that the handles are round (Fig. 5), and a trifle larger than any yet shown. I found during these experiments with handles that a sensitive touch, firmness in grasp, and accuracy in manipulation increased in proportion as I enlarged them. Might this not go on to a greater extent? I think not; the limit ceases when the cushions of the fingers can no longer vibrate with immediate contact with other portions of the hand.

Wood is pleasant to hold, light, and subdues a steely vibratory sound both upon the teeth and cabinet; I am adopting them both in excavators and gold-trimmers.

By its use we are enabled to select colors which will assist in quickly recognizing the points desired when they are thrown upon a bracket promiscuously, without tiring the eyes in searching for the smaller object, the point.

The butts of the handles point out at a glance the right from the left; and this simple suggestion assists materially in resting the eyes. I have searched carefully to get the most distinct colors, and out of all the varieties of natural wood have only been able to secure nine. Those at the right are ebony, then mora, leopard, and coccoloba. These are in pairs. The single ones are red cedar, mahogany, sycamore, and white holly, the last five having the same shaped butt, and are caught with the eye by the color only.

The cone-shaped ferrule that receives the point is a solid piece of metal; so shaped as to form a ferrule over the end of the wood, and the body extending for an inch and a half down the handle. This receives the point with a screw thread, and when turned in firmly never turns during the operating.

Before entering into the particular service of each point in the set, I will call your attention to a mathematical principle in shaping cavities that follows in our line of work with few exceptions. Let us look at this slide a moment (Fig. 6): these cuts are selected

Fig. 6.

from Dr. Ottolengui's and Dr. George Evans's methods of forming cavities; in these you observe that all the cavities and their retaining surfaces represent spheres or parts of spheres. This is true to the order of natural law for obtaining the greatest mechanical strength.

If we should take any normal tooth and divide it into sections, and continue its curves, we should form spheroidal shapes. In this figure (7) this corkscrew point likewise forms, by following its

Fig. 7.

peripheral curve, a sphere; the smaller circles that you see represent some portion of the point curvatures. You will also observe the form of bur I have used exclusively in my practice: these are round. Here we have three corrugated forms that apply to each other with sympathy of adaptability that coalesce strongly and practically. Let me now demonstrate the method of filling this tooth.

I will suppose that the cavity has sufficient retaining walls to hold a mature contour filling. At a convenient point at the cervical margin I should make a retaining groove; with a No. 1 round bur in this groove drill a pit for the starting-point, then fill it with hand-pressure until it is well out of the pit, and immediately begin to burnish piece after piece on its surface with the third pair of corkscrews; completing the entire filling with one pair of instruments only would be practical, but not necessary.

To increase the rapidity of the operation, after I was out of the retaining grooves, "which this point will follow with a nicety of adaptation that is very gratifying," I would complete the work with a flat burnisher.

Next, let me fill this upper or lower second bicuspid on the distal surface. (Fig. 8.) I should use crystalline surface soft gold-foil, No. 4, cut each sheet in thirds, roll into rope form between the thumb and finger, making a cylinder; this I pack in each side and key in the centre,—a very familiar practice with you all.

This would be condensed by the use of the same form of corkscrew, only two sizes larger, and deeply curved so as to reach any cavity in the mouth. These are used for packing soft foil, and are finely serrated to prevent slipping and to assist in the mechanical union that exists between unannealed and annealed foil.

You will picture in your minds the soft foil covering the cervical wall, and reaching up far enough to protect the danger-line where the enamel ceases. I only use one layer of soft foil.

Fig. 8.

I begin at once to anneal my foil, or any form of gold that is, as we term it, cohesive, and pack with hand-pressure along the right angle at the wall protecting the pulp. I have no difficulty whatever in starting my work here, and wonder at it myself.

I have now proceeded far enough to use the second size of corkscrew points, and begin to burnish the gold into the under-cuts down upon the soft foil, which stiffens the cervical body, and the polishing up and over the walls of this fragile portion of enamel surface with a comfortable reliance that has relieved me of all anxiety of its becoming disintegrated during the process. I continue with this point until I wish to begin to contour, when I would use a flat obtuse burnisher, the surface of which is at right angles with the handle, building the contour to any desired form or delicacy without having the least trepidation concerning its cleaving or breaking away.

The mesial cavities are filled in like manner, except that a foot burnisher is used for contouring.

The molars, bicuspids, and distal surfaces of cuspids are manipulated with these instruments.

In leaving this part of the topic I will show the improvements upon the size of handles and short-shanked points, and simply say that I cannot recall any instance where the large handle and short point interfered by being in the way while operating. (Fig. 9.) The other accessories necessary to complete the outfit for this form of working will be shown in this slide. (Fig. 10.) A doily is placed upon a bracket from which I pick up the gold for annealing, using the same burnishing point for that purpose, and the temper of the instrument has not suffered any loss by being used in this way.

Fig. 9.

Fig. 10.

The gold here represented is No. 3 foil, rolled in rope form and flattened out by passing a round-handled instrument across it. This is done simply to facilitate in passing it to the cavity, and does not diminish the cohesive quality. This flat piece is a serrating file, to assist in keeping a roughened point surface that is necessary for picking up the gold. Passing the file across the point of the instrument is sufficient to make the gold adhere to it. This disk holds a piece of crocus paper, which is used for polishing the burnisher; occasionally it adheres to the surface of the gold.

I have never been able to analyze this problem, and have questioned Professor C. R. Cross, electrician at the Institute of Technology, regarding it, and experimented along his line of thought without discovering any electrical disturbance.

The next important of all the appliances is this alcohol-lamp (Fig. 10), which I purchased at the dental depot. The shield was made for me, as a broader and larger protection was required for the flame; the inner surface being blackened so that the flame could be discerned more quickly.

I have annealed upon metallic trays with perfect safety, using gas, but never over a Bunsen burner.

Upon this matter let me use the words of Professor G. V. Black in his paper read before the New York Odontological Society.

"Gold-foil possesses the power of condensing or occluding gases and volatile substances upon its surface. That when such gaseous occlusion took place the welding property was entirely destroyed. In view of the readiness with which gold-foil absorbs gases, the use of a naked flame for annealing gold-foil is objectionable, for the reason that the foil is subjected to the action of the products of combustion, which in the case of a Bunsen burner contains, besides the usual water and carbonic acid gas, a greater or less amount of sulphurous anhydride, and should the imperfection of the combustion be in any way modified, acetylene and other deleterious hydrocarbons are produced." He further states, "If a naked flame is used, ninety-five-per-cent. alcohol, with asbestos wick, is the least objectionable."

This analysis of Professor Black is practically substantiated by Professor Thomas Fillebrown in his work on "Operative Dentistry," and he approves of annealing by the use of the alcohol flame. I have not discovered any objection in the use of the alcohol flame, passing the gold through it or a little above, being not over particular in this matter; sometimes it reddens and melts at the extreme thin portions, but it all welds, and the work goes on without disconcerting me in the least.

There is one more important device that I use for repairing gold filling. (Fig. 11.) This shows a glass tube that is packed with

Fig. 11.

cotton and saturated with alcohol, and when lighted the flame can be attenuated to any desired length, and at any angle, by the use of the chip-blower.

In undertaking to repair a gold filling, and after the surfaces are cleansed, I flash the flame across the surface to insure perfect safety. However, it is not as necessary in burnished work as in other forms of gold work, as it is less porous.

Now, dismissing this part of the theme, I will ask you to follow me through a series of experiments, taking up the various methods of manipulating gold, each having its admirers and advocates. On this slide (Fig. 12) is shown a block of ivory three inches long, two inches wide, and one-half inch thick, which is constructed in two sections, which are bolted firmly together at each end. In the centre you observe a fissure one inch long, with a depth sufficient to admit a pear-shaped finishing bur. Being impressed that if necessary caution were used I might arrive at some conclusion regarding their characteristics.

To follow out this thought by a practical test, Dr. Thomas B. Hayden kindly assumed the responsibility of filling the fissure.

Fig. 12.

My object in choosing him is that he was fresh from the hands of his instructors at the Harvard Dental School, and had not acquired any one method that would prejudice him.

In every case Dr. Hayden employed one manufacturer's gold, using No. 3 cohesive foil, rolled in rope form, with sheets divided in thirds. He began the work at the extreme end and continued in a diagonal manner until it was completed. The time was carefully noted, and his own good judgment dictated his procedure.

The six methods that he took up were labelled and submitted to the microscopist, Mr. H. S. Smith, who used every endeavor possible to produce the best results.

Those of you who have had any experience in photo-micrography will appreciate the difficulties that arise in trying to procure a comparatively clear positive from an opaque, reflecting object, and will acknowledge, I think, that he has been able to show very distinctly the imperfections of our gold work. We will

Fig. 13.

begin with the hand-mallet. In all of these slides you will discern light and dark patches representing the portions of the cavity that have been perfectly and imperfectly filled. This, I will admit, looks formidable, but if you comprehend the magnification being so great we will forgive the disclosures and the inventor of these powerful lenses.

In making this bar, Dr. Hayden used a Harvard foot-plugger, and I an eight-ounce lead mallet. The time required for filling this fissure was one hour and twenty minutes; weight, twenty-one

Fig. 14.

grains. In our next exposure of Dibble's pneumatic mallet, we meet with deeper depressions, an indication of less density, yet it shows an interlacing of its molecular structure that is interesting. Time required, one hour; weight, ten grains.

Fig. 15.

In this we represent the work of the Snow & Lewis automatic mallet. Here we have a very decided improvement over the hand- and pneumatic mallets, the texture more homogeneous, a quality that we are seeking for.

The fissures, or what is commonly called rat-holes, are less pronounced. Its contact with the surface of the cavity patchy, but comparatively good. Time of filling, two hours; weight, fourteen grains.

The work of the Bonwill mechanical mallet now appears upon the screen, representing a similar appearance, not quite so badly

Fig. 16.

distributed in masses as the automatic, otherwise apparently very dense in structure. This was filled in one hour and five minutes, weight twelve grains.

Fig. 17.

The next to follow is the hand-pressure. We are to judge of its merits through the centre, as we focussed at the most desirable portion of the bar.

After what has been discovered already, we are prepared to see a porous structure, with pits showing that serrated instruments produced them, and a cohesive interweaving of the lamina of gold.

Fig. 18.

Time of filling, one hour and thirty-five minutes; weight, thirteen grains.

The next illustration shows the burnishing method. In this we have less deep cavities and pits, more fibrous in structure and a surface adaptability, for which we are aiming. Time of filling this was one hour and thirty-five minutes; weight, fifteen grains.

Fig. 19.

The accompanying slide shows the result of my work under the same conditions; it is very gratifying to witness such results as you see. It has a fibrous appearance, homogeneous density, and adaptability that we would expect if a plastic material were burnished against dense walls.

What have we learned by this analysis? first, that the hand-mallet pounded more gold into a given space than any other method, and yet its object of condensing against its walls was far from being perfect.

In connection with this result, let me quote Dr. Ottolengui from his book on methods of filling teeth. These are his words: "The theory was that the more gold one could crowd into a cavity the better the result." He says this is not true, and states also the supreme demand upon any filling is "that it shall present a durable surface, and be in close contact with all its walls." I am convinced that he has the sympathy of the fraternity in this matter.

Of the Dibble pneumatic mallet you have already formed your opinions of its structure and weight.

The automatic mallet is the universal instrument for filling teeth. What physical phenomena do we observe under its treatment? In your gold work of all descriptions you have discovered an inherent propensity of the metal for becoming hardened and tempered, by hammering or malleting. In our laboratory work we redeem the soft elastic quality by repeated annealing.

Can we do this in malleting in a gold filling? Certainly not. Then what happens? We are constantly tempering and hardening the mass, making it more obstinate in conforming to our wishes, less elastic and fibrous, and less suited to our needs, as the slide plainly shows deep pits and grooves against its walls. Is the tempering and hardening a desirable feature towards perfect work? To my mind it appears only for one purpose, that of giving strength, an acquisition not to be ignored, but to be supplanted by a more humane and satisfactory way.

The references I make regarding the Bonwill mechanical mallet are from experienced operators. "Dr. Louis Jack refers to it as being rapid in movement, and that gold can be packed with great rapidity, and with very considerable density. The complaints of patients after they become accustomed to the great velocity are less than in most forms of effectual malleting."

"Dr. Ottolengui prefers it to other mechanical devices for operating, and the comparison he makes of patients' choice between the use of this and the hand-mallet is that fully ninety per cent. choose the Bonwill."

To solve the problem of hand-pressure I take great pleasure in referring again to Dr. Ottolengui, as I deem it a duty to those who are listening that an authority should be adopted that has had, as it appears, an unbiassed experience with all gold work. You will easily see that my knowledge is worthless in individual practice with any of the accepted methods. Therefore I will repeat after the doctor in his paragraph, "How to condense Gold." "The greatest good gained by hand-pressure is that gold remains more cohesive under this method than in connection with any other," saying "that he had sufficiently tested it to feel safe in making the following dogmatic statement: the more gradual the pressure exerted upon gold-foil, in condensing it, the less it loses its quality of cohesiveness, and, *vice versa*, the more sudden, sharp, or rapid the blow of the hammer, the less cohesiveness will be exhibited."

This whole chapter on "Gold as a Filling-Material" is deeply interesting. Referring to this case again, we place alongside of these remarks the observation revealed by the microscope, noting its texture and weight, and there must leave it with its admirers.

In taking up the methods of burnishing or welding gold, I find several very able writers who have touched upon this theory with a mystifying sense of doubt and temerity which has impressed the professional brotherhood with awe and hesitation. For instance, one of your very distinguished members, Professor Fillebrown, in a paper that was published in 1873, entitled, "Another Method of packing Gold," describes very minutely the details that are necessary to achieve success with smooth steel points, stating that the manner of applying the force must be a steady pressure with only a slight turn of the point; any rubbing of the gold must be avoided; it destroys the cohesiveness of the surface so that more cannot be added.

Would not directions of this kind alarm any operator, and make him cling all the closer to his early teaching? Dr. Ottolengui has resorted to the burnisher for contouring when teeth have become sensitive from prolonged malleting, with satisfaction to his patients and to himself; and he impresses you that you should not proceed any further.

Dr. Herbst used burnishing points, rotating them with an engine. Dr. Frank Abbott found no difficulty in cohering gold with smooth points.

Doubtless there are many in this audience who have burnished gold in some degree; and why is it to-day that dentists are afraid of undertaking this simple method of working gold? I think three words will explain it,—unsuitableness of instruments.

I have been unable to find any reliable information or reasonable excuses for not using suitable smooth steel points for inserting gold fillings by a burnishing process.

Now that the microscope and test of weight permit me to show enthusiasm without the appearance of egotism, you will allow me to speak freely. What has it done for me? First and foremost, my patients are benefited; their sufferings are lessened to at least one-half of the usual pain.

When the cavity is prepared the pain ceases, comparatively speaking; and what does this mean in itself? That I have patients from distant cities visit my office for the more difficult gold work, which they know can be done painlessly and satisfactorily. Furthermore, many delicate teeth filled with cement and gutta-percha have been treated successfully; likewise, a great number of fillings with discolored margins have been restored for better adaptability of gold to their frail enamel walls. Teeth that have been given up as lost are restored to beauty and usefulness.

In comparing notes with other operators I find another advantage in this very simple method that is prized both by patient and operator; the rapidity of the work is increased at least one-third. You all know the meaning of the distribution of forces, how the molecular structure of a tooth takes it up and passes it along, or if its impact becomes too intense, that it disintegrates the enamel prism.

It is understood by you all how sharp concussion acts upon any solid body, and you cannot fail to see that the same force applied slowly will be redisturbed without injury. I speak of it as being a simple method. What appears to be difficult about it when once you have the confidence I am unable to discover.

It is not necessary that you should have the instruments that have been shown you to-night. Improvise the flat burnisher,—any corkscrew, foot-point, or broken excavator. However, I would consider the handle, for it does require a comfortable amount of force to condense gold properly; pick up your gold with the point and anneal with it; as I said before, my gold rarely adheres to the point, possibly because it passes through the alcohol flame.

Spend a night or two experimenting, and you will be convinced that it is simple, trustful, and practical. After all this enthusiasm, the question may be properly asked, Why have you not let the profession learn of this before? I will tell you. First, I had not completed my experimental work with the instruments until within the last few years. Secondly, an opportunity which I deemed appropriate had not presented itself. I realized what it meant to the human family. I knew what it meant to the operator.

I dared not move until I was surrounded by encouraging sympathizers. This anxiety and doubt I explained to my friend Dr. Gerrish, of Exeter, New Hampshire, who urged me to show what I was doing. This was two and a half years ago. Still I hesitated; until meeting your president, six months ago, while talking the matter over with him my duty was made clear. He well knew the fear was due to the fact that there would be little good gained unless I had supporters to help establish its claims, and I would gladly have deferred this privilege even longer were it not for his kind entreaties.

Gentlemen, I knew eight years ago that I was having an experience worthy of consideration. I knew it possessed a merit and a quality that humanity is suffering for, and my motive for waiting was a far-reaching one; it enters the homes of a multitude of sufferers who do not visit us, having thoughts of our treatment. Then let us not be content, but try and inspire our patrons with the assurance of our sympathy and best endeavors.

## Figures and Tables

**Fig. 1. f1:**
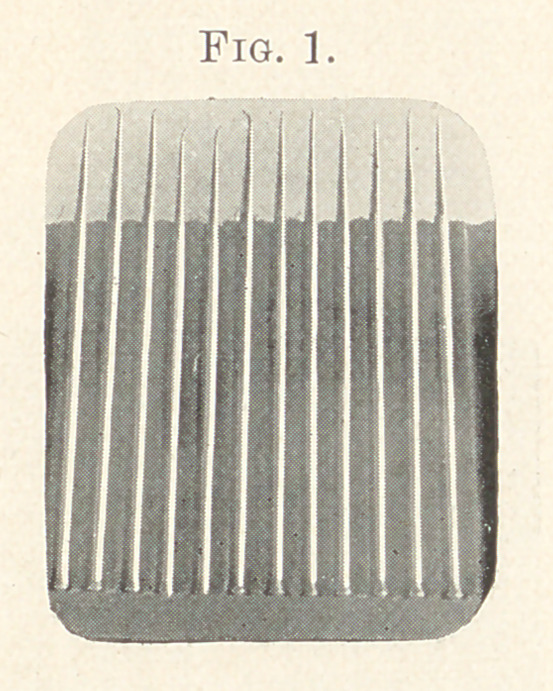


**Fig. 2. f2:**
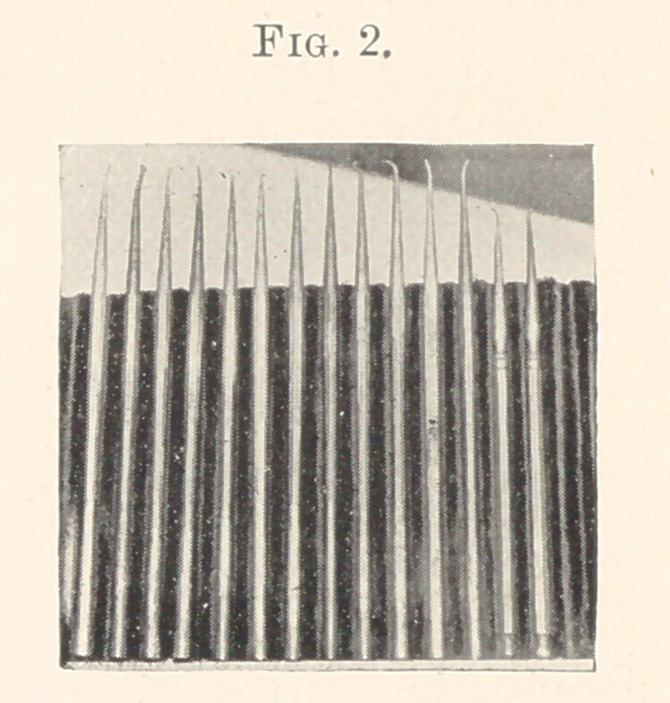


**Fig. 3. f3:**
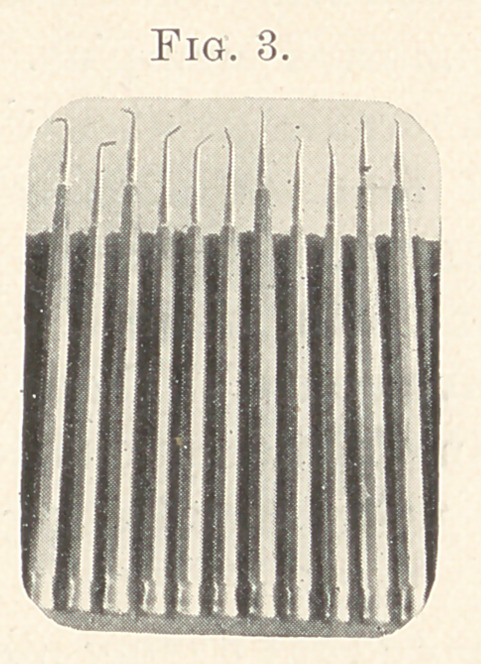


**Fig. 4. f4:**
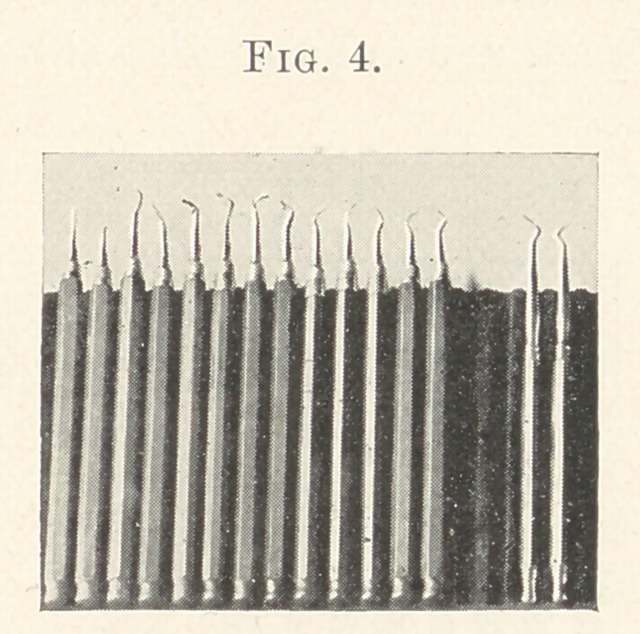


**Fig. 5 f5:**
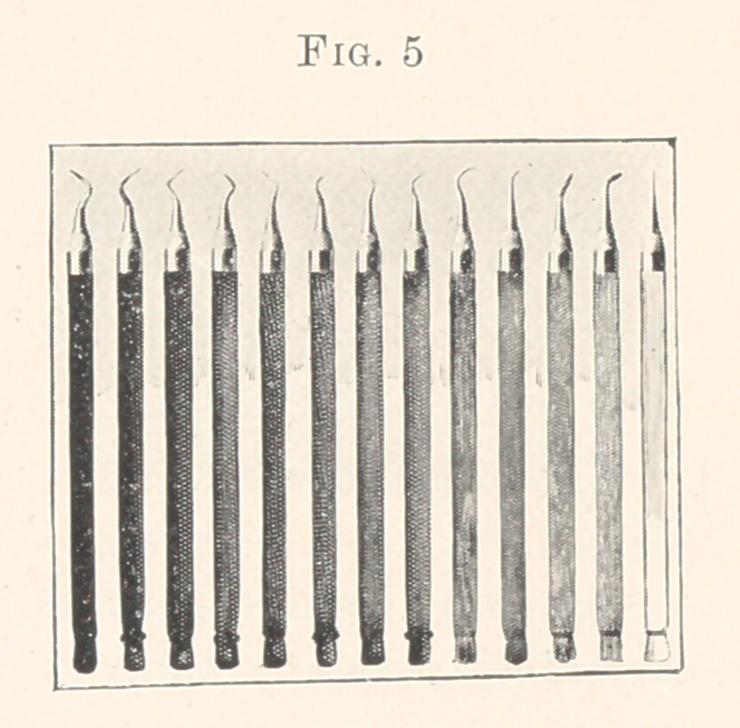


**Fig. 6. f6:**
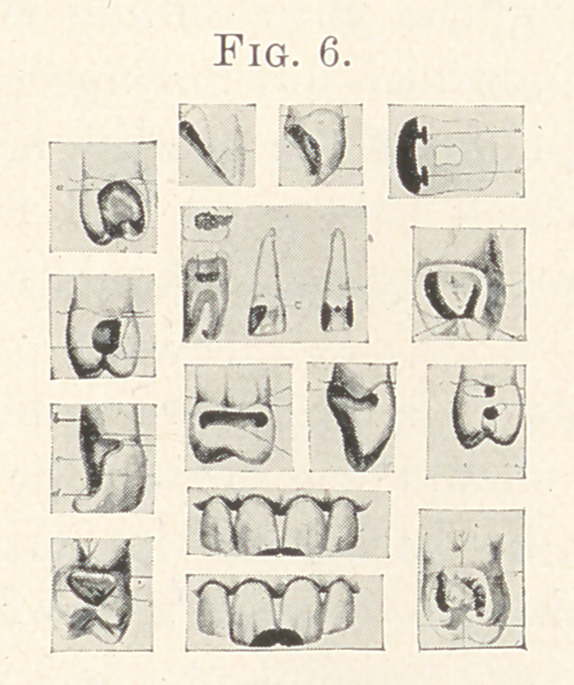


**Fig. 7. f7:**
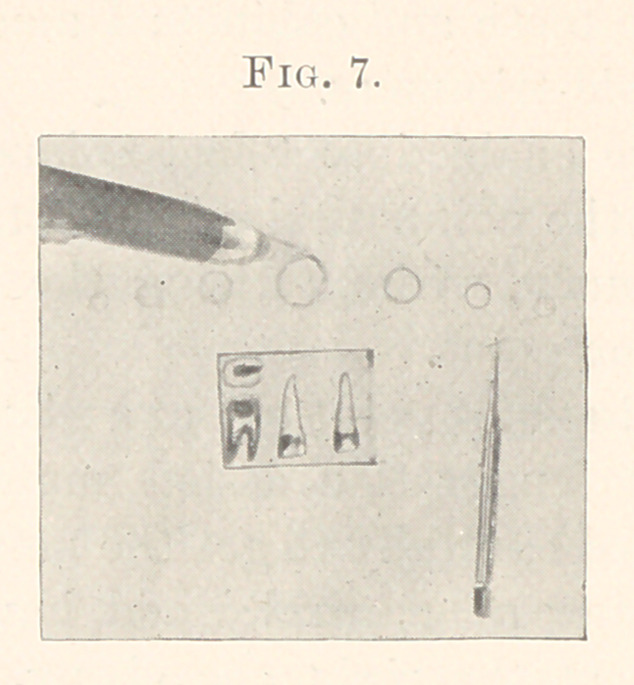


**Fig. 8. f8:**
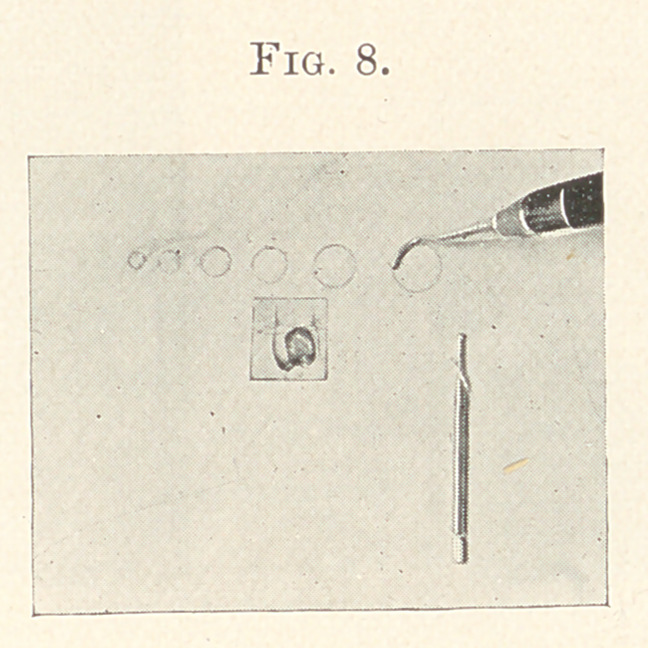


**Fig. 9. f9:**
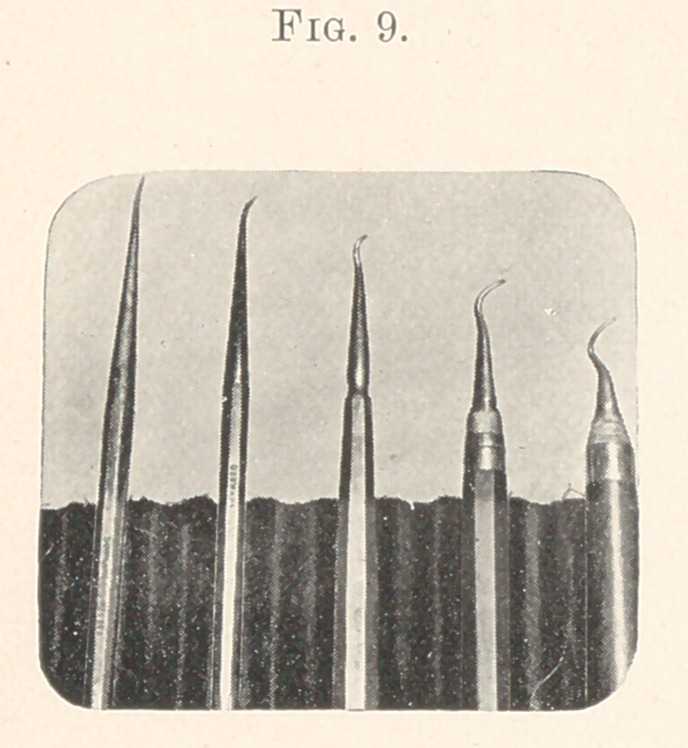


**Fig. 10. f10:**
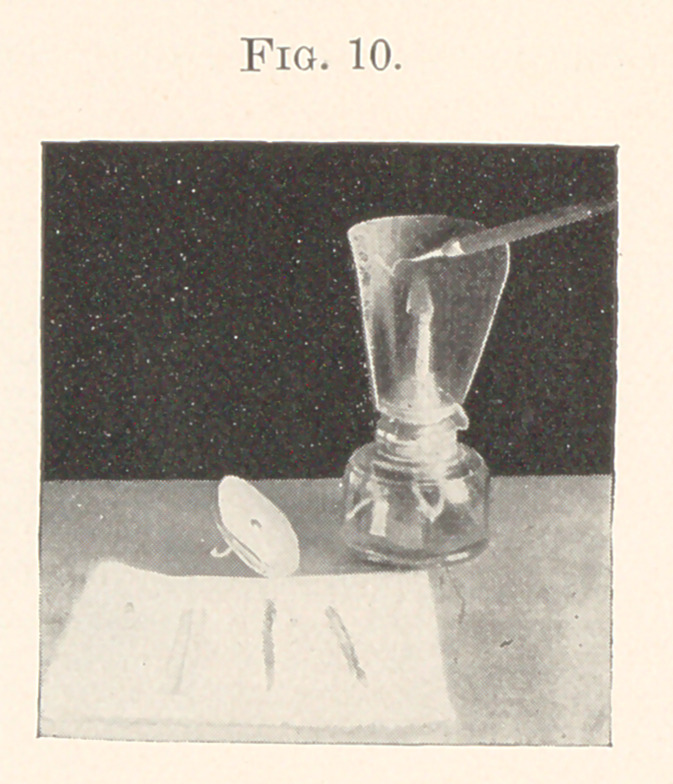


**Fig. 11. f11:**
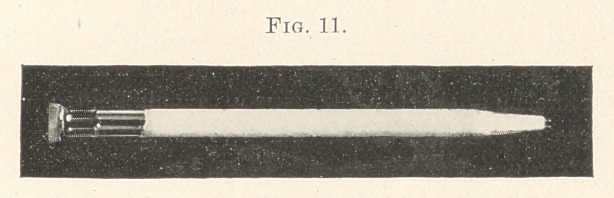


**Fig. 12. f12:**
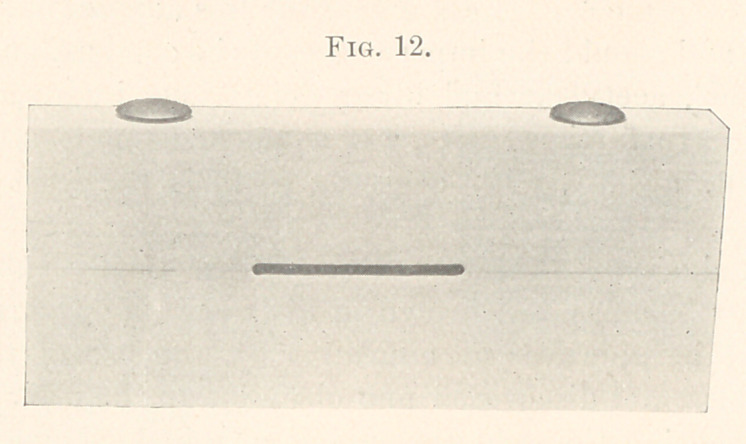


**Fig. 13. f13:**
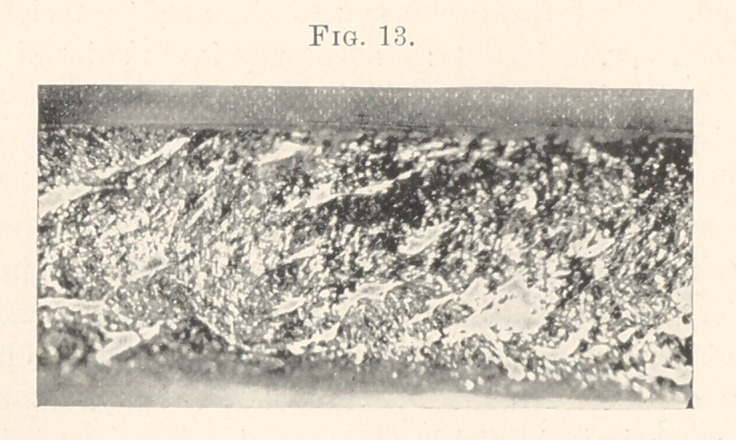


**Fig. 14. f14:**
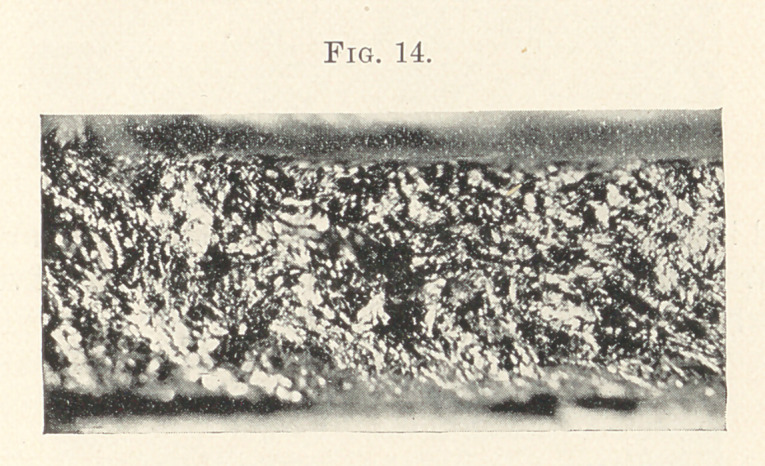


**Fig. 15. f15:**
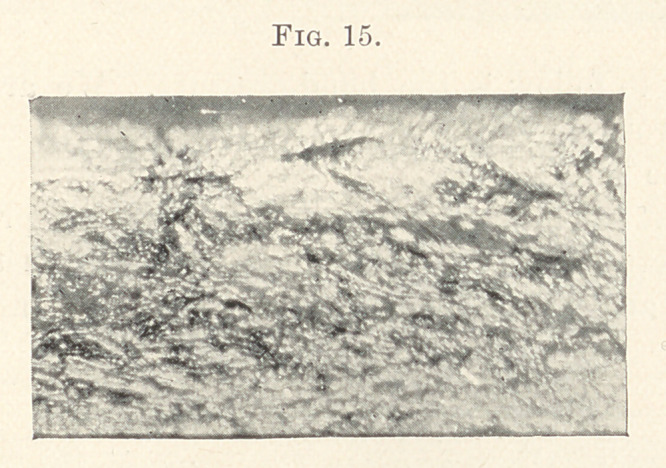


**Fig. 16. f16:**
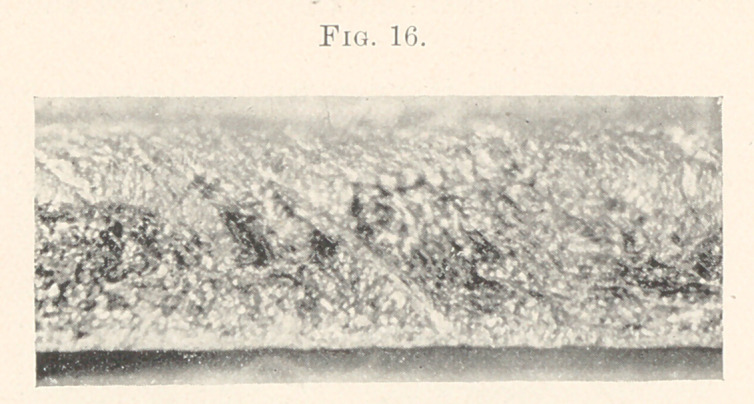


**Fig. 17. f17:**
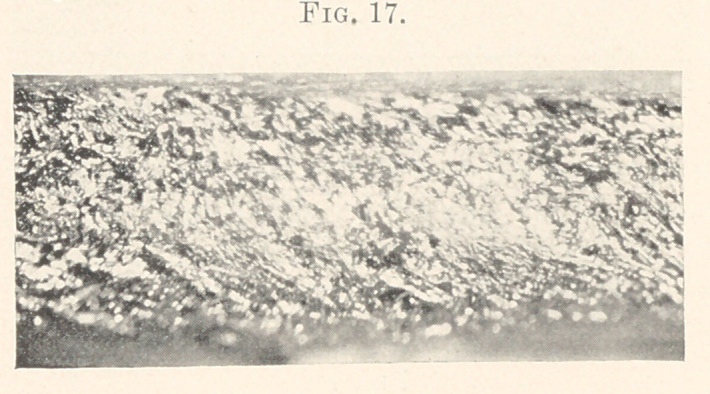


**Fig. 18. f18:**
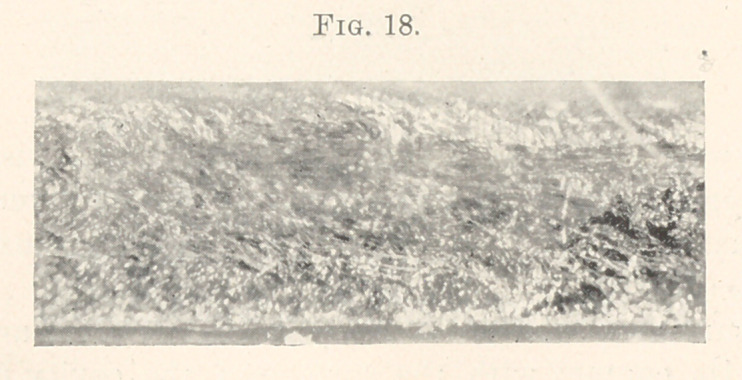


**Fig. 19. f19:**